# A fine‐needle aspiration‐based protein signature discriminates benign from malignant breast lesions

**DOI:** 10.1002/1878-0261.12350

**Published:** 2018-08-09

**Authors:** Bo Franzén, Masood Kamali‐Moghaddam, Andrey Alexeyenko, Thomas Hatschek, Susanne Becker, Lotta Wik, Jonas Kierkegaard, Annika Eriksson, Naveen R. Muppani, Gert Auer, Ulf Landegren, Rolf Lewensohn

**Affiliations:** ^1^ Department of Oncology and Pathology Cancer Center Karolinska Karolinska Institutet and University Hospital Stockholm Sweden; ^2^ Department of Immunology, Genetics and Pathology Science for Life Laboratory Uppsala University Sweden; ^3^ Department of Microbiology, Tumor and Cell Biology (MTC) Karolinska Institutet Stockholm Sweden; ^4^ National Bioinformatics Infrastructure Sweden Science for Life Laboratory Solna Sweden; ^5^ BröstCentrum City Stockholm Sweden; ^6^ Capio S:t Görans Sjukhus Stockholm Sweden; ^7^ KIGene MMK Neurogenetics Unit CMM Karolinska Institutet Stockholm Sweden

**Keywords:** breast cancer diagnosis, fine‐needle aspiration, protein biomarker, proximity extension assay

## Abstract

There are increasing demands for informative cancer biomarkers, accessible via minimally invasive procedures, both for initial diagnostics and to follow‐up personalized cancer therapy. Fine‐needle aspiration (FNA) biopsy provides ready access to relevant tissues; however, the minute sample amounts require sensitive multiplex molecular analysis to achieve clinical utility. We have applied proximity extension assays (PEA) and NanoString (NS) technology for analyses of proteins and of RNA, respectively, in FNA samples. Using samples from patients with breast cancer (BC, *n* = 25) or benign lesions (*n* = 33), we demonstrate that these FNA‐based molecular analyses (a) can offer high sensitivity and reproducibility, (b) may provide correct diagnosis in shorter time and at a lower cost than current practice, (c) correlate with results from routine analysis (i.e., benchmarking against immunohistochemistry tests for ER, PR, HER2, and Ki67), and (d) may also help identify new markers related to immunotherapy. A specific 11‐protein signature, including FGF binding protein 1, decorin, and furin, distinguished all cancer patient samples from all benign lesions in our main cohort and in smaller replication cohort. Due to the minimally traumatic sampling and rich molecular information, this combined proteomics and transcriptomic methodology is promising for diagnostics and evaluation of treatment efficacy in BC.

**Abbreviations**
BC: breast cancer
ER: estrogen receptor, ESR1
FNA: fine‐needle aspiration biopsy
HEPES: 4‐(2‐hydroxyethyl)‐1‐piperazineethanesulfonic acid
HER: HER2‐positive
HER: receptor tyrosine‐protein kinase erbB‐2, ERBB2, HER2
IHC: immunohistochemistry
Ki67: antigen KI‐67, Ki‐67, MKI67
LumA: BC subtypes luminal A
LumB: luminal B
LumHER: luminal HER2‐positive
NPX: normalized protein concentration
NS: NanoString
PEA: proximity extension assay
PR: progesterone receptor, PGR
RIPA: radioimmunoprecipitation assay buffer
TNB: triple‐negative

## 1 Introduction

Key challenges at the time of primary evaluation of a lump in the breast are the distinction between benign or malignant processes and, in the case of malignancy, the choice of optimal therapy. A significant number of breast cancer (BC) cases may be missed due to tumor heterogeneity not uncovered by current diagnostic procedures. Studies indicate that 15–40% of all patients with BC may be overdiagnosed or overtreated (Bell, 2014; Tofigh *et al*., 2014). Accordingly, there is an urgent need to implement new molecular tools to improve diagnostics and therapy selection.

Fine‐needle aspiration (FNA) sampling is a well‐established cancer diagnostic procedure in Sweden since the 1970s. Using FNA biopsy cells, tissue fragments and/or fluid may be recovered from tumor tissue via puncture using a thin‐gauge needle (21–25 G). The tip of the needle is placed in the center of a lesion, and cells are aspirated via a syringe during gentle oscillation back and forth. The minimally traumatic FNA is often used for diagnosis of small nonpalpable, deeply located, or otherwise hard‐to‐reach lesions, both for primary tumors and for metastases, if necessary assisted by ultrasound (US) (Ly *et al*., 2016; Rimsten *et al*., 1975). However, the efficacy of FNA largely depends on the experience and skill of both aspirators and cytopathologists. Further limitations of FNA sampling are the loss of tissue architecture for tumor grading, the inability to inspect the invasive front of the tumor, and the small amount of material available, which may limit downstream molecular or morphological analyses (Roy‐Chowdhuri *et al*., 2017).

FNA samples, including material leftover in the needle after preparation of samples for cytological examination, have proven useful for mRNA profiling via RT‐PCR to strengthen the diagnosis (André *et al*., 2009; Annaratone *et al*., 2012). This option for molecular analysis may be of value for several reasons: (a) Minute lesions detected by mammography provide limited amounts of postsurgery material for molecular analysis; (b) degradation or the molecular composition may occur during transport of surgical specimens to the pathology laboratory for sampling, while FNA material can be instantly preserved; and (c) leftover material in the needle matches the material used for cytology. If desired, additional FNA samples may be obtained from different locations to investigate heterogeneity.

Many studies use labor‐intensive methods and procedures for handling of material, extraction of RNA and molecular analysis, etc. Before introduction for routine use, sample preparation procedures need to be simplified to reach clinical utility. Our study explores molecular profiling of FNA samples, primarily at the protein level but also via RNA. In this study, two different technology platforms were selected for analysis of FNA cell lysates from leftover FNA‐needle material.

The first platform was protein profiling using proximity extension assays (PEA) (Assarsson *et al*., 2014). To date, only a small number FNA‐based multiplex protein analyses in cancer have been reported (Ullal *et al*., 2014). PEA is an immunoassay that enables profiling of sets of 92 proteins in minute amounts of biological material. In this method, two antibodies linked to oligonucleotides recognize each target protein. When bound to the same target molecules, a polymerase produces DNA reporter strands from two specific oligonucleotides, attached to the antibodies, and the products are quantified using microfluidic qPCR (Fluidigm^®^) as a measure of the amount of the target protein. The assay specificity is very high and remains so with multiplexing. In this study, the samples were analyzed using the ‘Immuno‐Oncology I’ and ‘Oncology II’ PEA panels (http://www.olink.com). The first panel allowed us to explore a wide range of immune‐related biomarker candidates such as chemokines and immune‐cell markers. The second panel included IGF1R, ERBB2 (HER2), ERBB3 (HER3), and other relevant proteins.

The second platform was mRNA expression profiling by NanoString^®^ (NS) technology (http://www.nanostring.com). NanoString's ‘nCounter Analysis System’ uses molecular ‘barcodes’ and microscopic imaging to decode and count transcripts from up to several hundred genes per sample (Geiss *et al*., 2008). We used the PAM50‐RUI CodeSet that includes probes for, for example, *ER, PGR, Ki67*, and *HER2 (ERBB2)*.

The underlying idea for this study was that molecular profiling of FNA samples may provide data for conclusive diagnosis and therapy selection for BC in a cost‐effective manner. In addition, quantitative molecular profiling may offer valuable insights in the phenotype of the tumor. Therefore, the main objectives of this study were (a) to explore whether the minimal leftover material from FNA is sufficient for both PEA‐ and NS‐based molecular profiling, (b) to examine the intrasample and interpatient variability of molecular profiles, (c) to perform a preliminary benchmarking *vs*. routine marker analyses by immunohistochemistry (IHC), and (d) to explore the possibility to identify protein signatures that correlate with key features of tumors.

## 2 Materials and methods

### 2.1 Patient samples

The study was approved by the Ethical Committee of the Karolinska Institutet, Stockholm (Dnr 2016/1432‐31/4). Female patients from the age of 18 years with mammography‐detectable lesions were invited to participate in the study after taking part of the project information and accepting the informed consent form. The patient cohort was part of the planned diagnostic examination, and the material was collected during a period of 6 weeks. FNA samples were obtained under ultrasound guidance by experienced radiologists using 21‐ to 22‐gauge needles, and after sampling for routine cytology, leftover materials from the FNA‐needles were processed immediately.

### 2.2 Sample preparation

#### 2.2.1 Cell collection

Samples were processed as follows: FNA‐needles were rinsed with 3× 50 μL ice‐cold RPMI‐1640 medium supplemented with 10 mm HEPES and protease inhibitors (Protease Inhibitor Cocktail tablet, Roche, No. 04693116001). Cells were pelleted at 2000 ***g*** for 30 s, frozen on dry ice, and stored at −80 °C until approval and quality control by cytology. Total processing time was 2–4 min per sample. The size of pellets and levels of blood contamination/hemolysis were estimated using an arbitrary 4‐grade scale. Up to three samples per patient were collected, and each sample was assigned a consecutive FD# sample code. On three occasions, two sequential *ex vivo* FNA samples (i.e., ‘biological replicates’) were collected after surgery from three patients (#102, #118, and #145), 4–5 weeks after the first diagnostic FNA sample (FD24, FD70, and FD59).

#### 2.2.2 Preparation of cell lysates

Samples approved by cytological examination and with cell pellets of at least 0.5 μL were thawed on ice and lysed in 13 μL RIPA buffer (Sigma, Stockholm, Sweden; R0278) per μL cell pellet supplemented with protease inhibitors and a RNase inhibitor (Sigma, R1158). After mixing and lysis on ice for 15 min, debris was removed by centrifugation at 13 000 ***g*** for 15 min. One‐microliter aliquots of supernatant were used to measure total protein concentrations using Micro BCA™ Protein Assay (ThermoFisher Göteborg, Sweden; Kit No. 23235) and RNA concentrations using the Qubit^®^ RNA BR Assay Kit (ThermoFisher; No. Q10210) on a Qubit^®^ 2.0 Fluorometer according to the manufacturer's instructions.

### 2.3 Subtype classification

Routine core needle biopsy (CNB, 14‐ to 16‐gauge needle) tissue samples from patients with BC, acquired in parallel with the FNA samples, were used for IHC analysis of estrogen receptor (ER/ESR1), progesterone receptor (PR/PGR), the proliferation marker Ki67 (MKI67), and HER2 (ERBB2), according to routine guidelines. Classification of molecular subtypes was based on recommendations according to the St Gallen classification system (Goldhirsch *et al*., 2011). The cutoffs were defined in the Swedish National Guidelines for treatment of BC (Nationella Vårdprogrammet för bröstcancer, version 2.0, SweBCG 2018, in Swedish: http://www.swebcg.se/wp-content/uploads/2016/09/Nationellt-v%C3%A5rdprogram-Br%C3%B6stcancer-2018.pdf) and the Quality and Standardization Committee (KVAST) of the Swedish Society of Pathology (2018, in Swedish: https://medlem.foreningssupport.se/foreningar/uploads/L15178/kvast/brostpatologi/KVASTbrostcancer2018.pdf). The molecular subtypes were defined according to the following criteria: ‘Lum A’, luminal A‐like (ER‐positive and/or PR‐positive, i.e., more than 10% positive cells, low Ki‐67, i.e., less than 25% positive cells and HER2‐negative); ‘LumB’, luminal B‐like HER2‐negative (ER‐positive and/or PR‐positive, and high Ki‐67, i.e., more than 25% positive cells, and HER2‐negative, i.e., 0 or 1+ according to IHC); ‘LumHER’, luminal B‐like HER2‐positive (ER‐positive and/or PR‐positive, any value for Ki‐67, and HER2‐positive, i.e., 2+ or 3+); ‘HER’, HER2‐positive, nonluminal (ER‐negative, PR‐negative, any value for Ki‐67, and HER2‐positive, confirmed by HER2 amplification using routine FISH technology when IHC is 2+ or 3+); and ‘TNB’, triple‐negative (ER‐ and PR‐negative, HER2‐negative, and any Ki‐67).

### 2.4 Protein profiling by proximity extension assays

Samples were diluted with RIPA buffer to a total protein concentration of 0.5 μg·μL^−1^. One microliter sample per panel was analyzed by Proseek Multiplex Immuno‐Oncology I and Oncology II panels (Olink Proteomics, Uppsala, Sweden) according to the manufacturer's instructions and as described previously (Larsson *et al*., 2015). Each panel consists of 92 protein assays and four controls. Results were exported from the Biomark reader and normalized using Olink Wizard for GenEx software for further statistical data analysis (http://www.olink.com).

### 2.5 mRNA profiling by NanoString technology (PAM50)

Gene expression profiling was performed on the nCounter^®^ Analysis System with FLEX configuration using the research‐use‐only PAM50 CodeSet (NanoString Technologies, Seattle, WA, USA). The PAM50 CodeSet includes gene‐specific probe pairs to the PAM50 targets, eight housekeeping genes (*ACTB*,* GUSB*,* MRPL19*,* PSMC4*,* PUM1*,* RPLP0*,* SF3A1*, and *TFRC*), six exogenous positive control RNA targets, and eight exogenous negative control sequences. The hybridization reactions were performed according to NanoString Technologies’ procedures, with the exception that 75 ng RNA was added per reaction. Maximally, 2.2 μL of RIPA buffer sample lysate was added to each 30 μL hybridization reaction. Samples were hybridized at 65 °C for 24 h using a benchtop thermocycler with a heated lid set to 70 °C. The nCounter Prep Station and Digital Analyzer were run according to the manufacturer's specifications (http://www.nanostring.com). Results were normalized, processed, and delivered in Excel spreadsheet format for downstream data analysis.

### 2.6 Statistics

Quality control and data preprocessing (including normalization) of PEA data and NanoString/PAM50 data were made according to the manufacturer's recommended procedures.

We rendered all the expression profiles normally distributed using log_2_ of PEA and NS expression values. Next, in analyses involving phenotype variables of categorical or nonstandard distributions, we either log‐transformed the values to make them normally distributed (for Pearson linear correlation and ANOVA) or applied nonparametric (rank) statistics, as indicated in the respective results. The analysis was performed in R environment, where we also used the package glmnet for the lasso‐and‐ridge regression method (available from http://web.stanford.edu/~hastie/glmnet/glmnet_alpha.html) under α = 1 and other parameters set to their defaults. In correlation analyses of expression between pairs of proteins, we adjusted respective *P*‐values by Benjamini–Hochberg correction (Benjamini and Hochberg, 1995).

## 3 Results

### 3.1 Patient cohorts

We analyzed in total 38 samples from 33 patients with benign lesions and 34 samples from 25 patients with cancer. All samples, except postsurgery samples, are listed in Table S1. Patient and sample information is summarized in Tables S2 and S3 for patients with benign and malignant lesions. For an overview and IHC subtype assignment of the available cancer samples (Table 1).

**Table 1:** Overview of cancer samples. Classification of molecular subtypes was based on recommendations of the St Gallen classification system (Goldhirsch *et al*., 2011). For details, see section 2.3. In addition, rebiopsy samples from four patients were analyzed (Table S3)

No. of patients with cancer	Subtype class based on IHC (St. Gallen)	Grade	No. of samples for PEA	No. of samples for PAM50	No. of patients with multiple^a^ samples (*n* = 2)	No. of patients with multiple^a^ samples (*n* = 3)
9^b^	Luminal A (LumA)	I–II	14	6	3^c^	1
4	Luminal B (LumB)	II–III	5	4	1	
3	Nonluminal HER (HER)	III	5	5	2^c^	
5	Luminal HER (LumHER)	II–III	6	5	1^c^	
4	TNBC (TNB)	II–III	4	2	0	
Total: 25			34	22	7	1

^a^Samples from multifocal lesion, one FNA sample per lesion. ^b^Two of nine patients were diagnosed with lobular cancer. All others had ductal cancers. ^c^In three patients, samples were obtained from a primary tumor and from axillary metastases.

In total, 92 consecutive leftover samples (needles) were initially processed to extract the remaining material. However, it is not possible to know at the time of sample collection whether representative or sufficient material for molecular analysis has been recovered. Moreover, as in this study we limited ourselves to investigating the small amounts leftover material present in the needle, eighteen of the samples were later excluded from molecular analysis because they failed to meet the inclusion criteria. After PEA analysis, two additional samples were excluded (for an overview, see Fig. S1).

### 3.2 Protein profiling by PEA technology, success rate, and limits of detection

Only two cancer samples showed very low cellularity according to examination by microscopy. The total protein amount of sample FD58 was only 19 μg. After rebiopsy (11 days after the first FNA sample), 180 μg was obtained (sample FD79). The sample with the second lowest total amount of protein was FD53 (40 μg of total protein), obtained from a 10‐mm metastasis. To explore the feasibility of using PEA to analyze low‐abundant proteins (e.g., IL‐2, IL‐7, IL‐19, IL‐35) in minute amount of leftover FNA material, we calculated the fraction of missing protein expression data in each sample as the percentage of samples that were below the limit of detection (LOD) for a given protein. In total, 171 proteins were analyzed. The concentrations of most proteins in the PEA panels were above the LOD, and in total, 124 proteins showed less than 25% missing values across all samples (Fig. S2). For example, ERBB2 was detected 2.43‐ to 1034‐fold above the LOD in 78 of 79 samples. Overall, we found a weak but significant correlation between success rate of PEA measurements (i.e., numbers of samples with PEA data above the LOD) and pellet size (Spearman rank *R* −0.38 and −0.23, *P*‐values 0.0004 and 0.0364 for the Immuno‐Oncology I and Oncology II panels, respectively). The correlation of success rate with total protein amount was even weaker (Spearman rank *R* −0.27 and −0.12 with *P*‐values 0.0098 and 0.2808 for the two panels, respectively). Despite the very high assay sensitivity, we concluded that due to practical aspects of sample processing, at least 20 μg of total protein per sample should be available for optimal PEA profiling.

### 3.3 mRNA profiling by NanoString technology

The choice of cell lysis buffer was evaluated for compatibility to both PEA and NS analyses. RIPA buffer containing NP‐40 produced higher signals compared to RIPA buffer containing Triton‐X, and a hybridization solution with 10% RIPA NP‐40 buffer was found to be tolerated by the nCounter^®^ Analysis System (data not shown). In total, 24 BC samples (including two postsurgery FNA samples, FD97 and FD98) were selected and 75 ng RNA per sample was used for RNA profiling by PAM50.

### 3.4 Quality control (QC)

Protein and mRNA are sensitive to degradation in clinical samples. To minimize degradation, rapid and cold processing with the addition of protease and RNase inhibitors was used. Each sample was also checked for being representative and of sufficient quality by cytology evaluation. Nonrepresentative samples without tumor cells were excluded in general, but with a few exceptions: (a) sample FD58 from patient #103 contained some cellular material, but smearing artifacts led to the decision to perform a rebiopsy after 11 days (sample FD79) to obtain material for diagnosis by cytology. (b) FNA sample FD82 from patient #123, originally evaluated as a suspected lymph node metastasis, was finally diagnosed as healthy tissue.

The PEA analysis is known to tolerate hemolysis well (http://www.olink.com/products/document-download-center/#validationdata). Spiking tests of blood into cell culture samples indicated that the maximum level of hemolysis was generally well below 10% (data not shown) and that high contamination according to cytology was a sufficient criterion for exclusion of samples from PEA analysis (this applied to only one sample, FD42).

### 3.5 Analysis of biological replicates and intra‐ and interpatient variability

**Figure 1:**
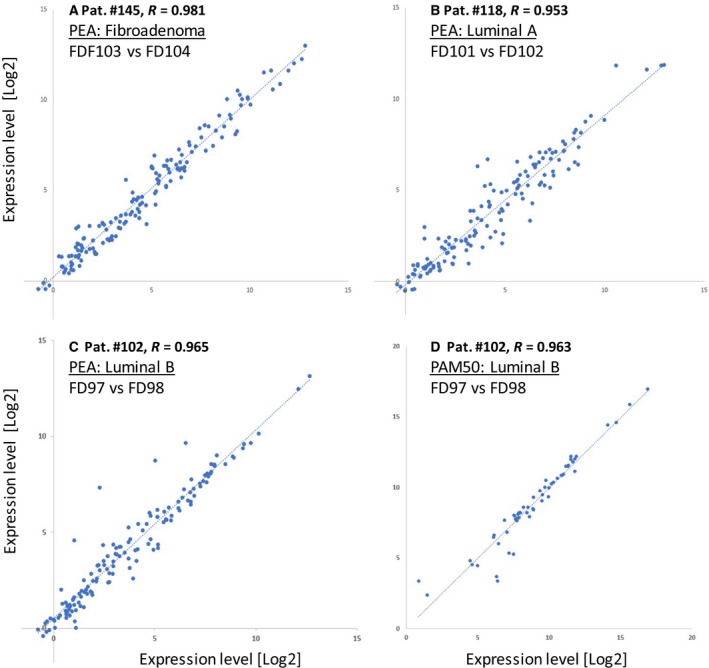
Comparison of expression profiles between duplicate postsurgery *ex vivo* FNA samples. Scatter plots show all normalized protein (A–C) and mRNA (D) values) that were above the limits of detection (LOD). Biological duplicates (two different FNA samples obtained *ex vivo* from the same lesion and then independently processed and analyzed in parallel) are plotted pairwise along the *X*‐ and *Y*‐axes. Expression levels of each protein are reported as normalized protein concentration (NPX values) in a 2‐log scale. Protein profiles exhibited high similarity between the biological replicates (average *R* = 0.966).

To identify differences between tumors with respect to expression levels, we explored biological replicates taken from the same location, and variability between different locations in the same patient or samples obtained pre‐ and postsurgery (tumor location identified by pathologist), as well as correlation between results for different patients with the same diagnoses. The technical reproducibility of PEA and of mRNA analysis by NanoString technology had been reported by the two providers to be very good, with CV < 10%. In both PEA analysis and PAM50 profiling, *ex vivo* FNA samples of postsurgery tumor material from three patients (#145, #118, and #102) demonstrated high similarity of expression profiles between repeated samples, with an average correlation (R) of around 0.97 (Fig. 1A–C). These patients were the only ones in our cohort that were subjected to surgery within the given period, and they did not receive neoadjuvant therapy. The material was compared to fresh cytological samples from the same three patients taken 28–35 days before surgery, permitting longitudinal analysis. The correlation values ‘presurgery *vs*. postsurgery’ for PEA profiles in patients #118 and #102 dropped to an average of 0.79 comparing all proteins with expression levels above the LOD. In addition, a marked shift of the slope indicated that the levels of many proteins increased after surgery. For instance, many immune‐related proteins (e.g., CD8A, CD5, IL‐8, CCL4, CXCL9, MMP12) showed greater than 10‐fold increases. In contrast, the correlation between PEA profiles of the fibroadenoma samples was considerably higher, that is, *R* = 0.95, but protein levels were relatively unchanged (Fig. S3a–f). The ‘cold ischemia time’ for all three specimens was 30–40 min, which together with effects of tumor progression may account for the altered protein levels after surgery.

Sampling of multifocal lesions from a given patient was always performed on the same occasion, and samples from multifocal lesions were obtained from eight patients with cancer in total (Table 1). Interestingly, the correlation of PEA profiles between multifocal lesions (intrapatient variability) from two patients with cancer (#110 and #105, with luminal A and luminal B subtypes, respectively) was higher (average *R* = 0.92) compared to correlation between pre‐ *vs*. postsurgery samples (Fig. S4a–f). In contrast, analyses of samples from different luminal A or B cancer patients, that is, interpatient variability within the same cancer type, showed radically lower correlation levels (average *R* = 0.71).

In conclusion, global correlation analysis of PEA expression profiles demonstrated a very good concordance between biological replicates but differences between breast tumors that spanned from high correlation between multifocal fibroadenomas and multifocal luminal A and B cancers in the same patient, to significant variability between different patients with cancer, and also between pre‐ and postsurgery samples.

### 3.6 Benchmarking *vs*. routine IHC analysis of CNB samples

One major objective of this pilot project was to build confidence for FNA‐based measurement of key IHC markers in BC. For benchmarking purposes, CNB samples obtained directly after FNA sampling were analyzed by routine IHC for expression of ER, PR, Ki67, and HER2. In two cases where no CNB samples were available, surgical biopsy samples were used for IHC. Of these markers, only HER2 (ERBB2) could also be assessed using both PEA and PAM50 panels. Expression of ER, PR, and Ki67 was recorded by PAM50 only as assays for these markers were not part of any PEA panel.

Note that we did not use PAM50 for BC subtyping because the algorithm for this purpose is based on formalin‐fixed paraffin‐embedded (FFPE) tissue and not FNA samples.

**Figure 2:**
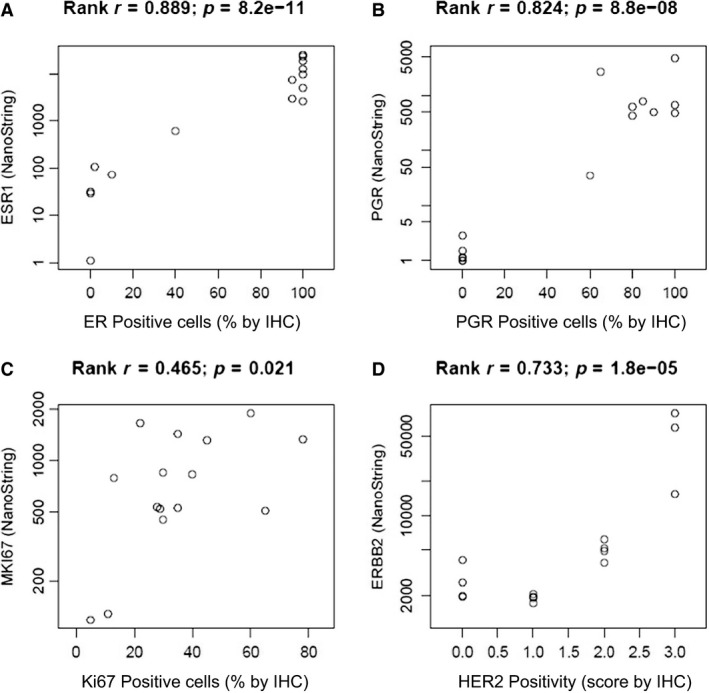
Comparison between mRNA and IHC analyses. Correlation between mRNA levels and IHC results for (A) ESR1 (ER), (B) PGR, (C) MKI67 (Ki67), and (D) ERBB2 (HER2). mRNA levels were analyzed using NS technology in FNA samples, while IHC was conducted in the corresponding CNB samples. The *X*‐axes of the scatter plots show the percentage of immune‐positive cells for ER, PGR, and Ki67, and level of HER2 positivity (0 to +3) given by the routine IHC report. The *Y*‐axes show log‐transformed mRNA expression levels (counts).

Results for all four markers showed a significant correlation between mRNA levels in FNA samples and IHC‐based data from the corresponding cancer tissues. Spearman rank correlation values were 0.889 for ER, 0.824 for PR, and 0.733 for ERBB2 (Fig. 2A, B, D). A lower Spearman rank correlation (*R* = 0.465, while still significant at *P* = 0.021) was obtained for Ki67 (Fig. 2C), which may be due to previously reported low robustness of IHC‐based Ki67 estimates (Sinn *et al*., 2017). These results represent the first evidence for the value of NS‐based mRNA analysis of key BC markers in crude cell lysates of fresh FNA samples.

### 3.7 Correlation between protein and mRNA expression levels of ERBB2

**Figure 3:**
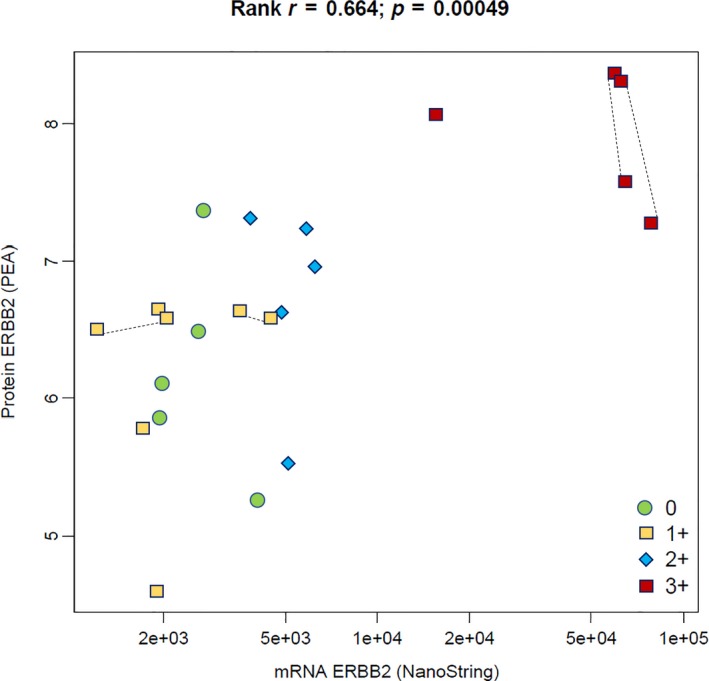
Correlation between protein and mRNA levels for ERBB2 in FNA samples. High values for both protein and mRNA measurements correlated well with HER2 IHC = 3+. Samples representing HER2 IHC = 0 or 1+ exhibited wider ranges of protein and mRNA levels. Lines between four pairs of symbols connect duplicate samples from the same patients, demonstrating high similarity between multifocal lesions, thus indicating consistent intrapatient levels. The *X*‐axis of the scatter plot shows normalized mRNA expression levels (counts) on a log scale, analyzed by NS technology, while protein expression levels of ERBB2 on the *Y*‐axis are reported as normalized protein concentration (NPX values) on the 2‐log scale.

ERBB2 (HER2) is a key biomarker determining the choice of therapy in BC, and FNA‐based analysis may be of substantial clinical utility. Therefore, we analyzed protein and mRNA levels in parallel. A significant correlation was observed between ERBB2 expression profiles in BC samples assessed by PEA and NS (Fig. 3). Data for ERBB2 showed that samples from patients with IHC score 3+ (amplification confirmed by FISH analysis) had on average twofold higher protein levels and 10‐fold higher mRNA levels compared to samples from patients with IHC score 2+. Protein levels of ERBB2 were also strongly correlated with ERBB3 protein levels (Spearman rank *R* = 0.92), which is in support of ERBB2/ERBB3 heterodimerization. Furthermore, multiple samples from the same individuals (*n* = 4) had very similar levels of both ERBB2 protein and mRNA.

### 3.8 Cluster analysis of PAM50 profiles

**Figure 4:**
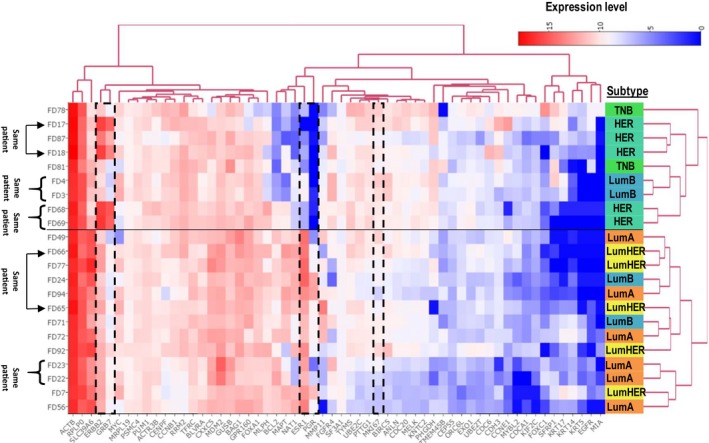
Clustering of PAM50 profiles. Unsupervised hierarchical clustering of samples based on PAM50 profiles demonstrates good correlation between IHC subtypes and key mRNA biomarkers. The *X* dimension represents transcripts and *Y* represents samples. We noted that five pairs of samples clustered close to each other within the two main sample subtype clusters. The horizontal line marks the separation of the two main sample clusters. Three of the five pairs of samples were immediately adjacent neighbors, and the other two were somewhat separated, one of which represented primary cancer and a lymph node metastasis (FD65 and FD66) and the other represented a multifocal, HER2‐amplified, and rapidly proliferating cancer (78% Ki67 IHC‐positive cells). Our interpretation of results is that the clustering pattern of samples underscores the technical reliability and biological validity of the FNA‐based expression profiling. All samples from the three patients with HER2‐amplified cancer showed highest levels of ERBB2 mRNA. This is illustrated by a dashed box together with GRB7, the mRNA expression of which is known to correlate with ERBB2. For comparison, additional boxes show expression levels of ESR1/PGR1 and MKI67, representing frequently used markers for IHC‐based subtyping of BC. An interactive representation providing data values can be explored at: http://research.scilifelab.se/andrej_alexeyenko/downloads/PEA/heatmap.PAM50.5subtypesXPAM50.v1.html

To further evaluate the reliability of FNA‐based mRNA profiling, we used cluster analysis of 22 samples (two postsurgery samples were excluded; see Fig. 4). The PAM50‐based subtyping of BC has previously been described using formalin‐fixed material (Nielsen *et al*., 2014) and approved by FDA. Several of the mRNA within the PAM50 profile are key markers for molecular BC diagnostics, for example, *ESR1, PGR, ERBB2, MKI67, EGFR, KRT5, KRT14,* and *KRT17*. In combination with other selected mRNA, the so‐called Parker algorithm may be used to separate BC into four molecular subtypes (luminal A, luminal B, HER2, and basal‐like) (Parker *et al*., 2009). In this study, we used the NS technology‐based PAM50 kit primarily for benchmarking key markers one by one, as described above. We also used the PAM50 data on 22 FNA samples for unsupervised hierarchical clustering to explore the correlation between subtypes, samples, and mRNA.

The clustering solution showed a good correspondence to the subtypes inferred by IHC (Fig. 4). Indeed, one of the two clusters represented more aggressive subtypes (HER and TNB, nine samples), while the other cluster included 13 samples representing less aggressive subtypes, including all samples with the luminal A subtype. We also observed that five pairs of samples representing multifocal lesions clustered more or less close to each other within the two main subtype clusters. However, one of three luminal B patients (represented with two samples, FD3 and FD4) clustered together with the more aggressive cases. The cancer from this patient had higher Ki67 levels and lower ER/PGR levels compared to the other two luminal B patient samples. This result is not surprising given the well‐known heterogeneity within the luminal B cancer subtype.

In summary, we found that (a) the observed clusters seem to reflect the expected subtypes and mRNA biology and (b) pairs of samples from individual patients tended to cluster close together. The cluster analysis thus strengthens our confidence in results of molecular profiling of FNA samples.

### 3.9 Protein‐based prediction modeling of cancer *vs*. benign lesions

**Figure 5:**
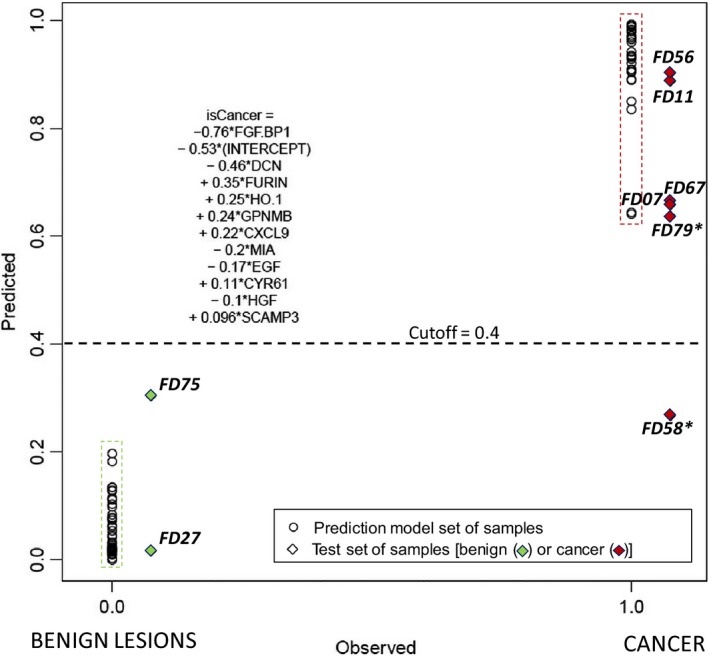
Multiple regression modeling of PEA data produced a signature for discrimination between cancer and benign lesions via protein levels. ‘Observed’ denotes the final conclusive diagnosis for each of the samples at a binary scale (0/1, *X*‐axis), and ‘Predicted’ is the quantitative score ‘isCancer’ assigned by the algorithm of the same range (0–1) but at a continuous scale (*Y*‐axis). Member proteins in the signature are described in the text. The predicted score for a given sample is calculated as a sum of protein expression values multiplied by the indicated coefficients. Circles represent samples in the training set and diamonds represent those of the test set, also identified by the FD‐sample numbers. Sample numbers with (*) are from patients for whom two samples were analyzed: Sample FD58 was discarded after cytology examination, and a new sample, FD79, was taken 11 days later. All patients in the test set were thus classified correctly according to this algorithm.

At this stage of the study, given the reproducibility and correlations between IHC and molecular analysis, we explored the possibility to identify key protein signatures. We summarized differences between cancer and benign lesions as protein signatures through multiple regression modeling. PEA protein levels were compared between benign and cancer lesions using cross‐validated multivariate modeling of PEA data *vs*. the final conclusive diagnosis (Fig. 5). We applied the lasso‐and‐ridge regression algorithm, which can compress the available data space via a cross‐validation procedure. More specifically, it retains in the model those protein variables that most significantly and most uniquely correlate with a given phenotype.

The algorithm identified an 11‐protein signature that appears to completely discriminate benign samples from malignant lesions. The algorithm yielded zero‐false positives and zero‐false negatives at a cutoff around 0.4. This protein signature (shown in Fig. 5) was selected from 124 protein variables in the two panels with the requirement that each protein to be stably expressed in the samples (i.e., proteins with expression levels below LOD in more than 25% of the samples were excluded). As the protein values, normalized according to the manufacturer's instructions, were within the same range, the absolute values of the coefficients indicated the terms’ significance. We conclude that the following top six proteins represented around 80% of the predictive power of the signature: (a) FGF‐BP1, fibroblast growth factor‐binding protein 1 (Q14512), linked to, for example, cell proliferation and cellular response to stress; (b) DCN, decorin (P07585), linked to, for example, vascular/tissue remodeling; (c) FUR, furin (P09958), linked to, for example, cell motility, extracellular matrix organization and proteolysis; (d) HO‐1, heme oxygenase (P09601), linked to, for example, heme catabolic process, DNA damage, and stress; (e) GPNMB, transmembrane glycoprotein nonmetastatic B (Q14956), linked to, for example, cell adhesion and cell differentiation; and (f) CXCL9, C‐X‐C motif chemokine 9 (Q07325), linked to, for example, promotion of tumor immunity and chemotaxis (gene ontology, biological processes). In addition, the levels of FGFBP1, DCN, FUR, and CXCL9 differed significantly between the benign and cancer groups by univariate analysis (ANOVA). Furthermore, levels of DCN and CXCL9 also differed between cancer subtypes (Fig. S5).

Naturally, the identified signature requires independent validation. As a first approach to that, we tested the PEA signature using eight samples from patients where the initial cytological examination did not provide a conclusive diagnosis (Table S3). A result that was considered conclusive was obtained for these patients a few weeks later, after additional histological and IHC examination of CNB samples (Tables S2 and S3). These samples therefore were not used in the training set for defining the signature. We found that all test samples in this limited replication cohort were correctly classified using the same PEA protein panel signature, with the exception for one sample (FD58). Sample FD58 was discarded after cytology due to insufficient amount of material, while a new sample, FD79, was taken from the same patient 11 days later, and this sample was correctly classified as cancerous. Thus, the signature enabled correct classification of all quality assured samples from the patients that initially obtained an inconclusive diagnosis.

## 4 Discussion

Our study demonstrates that lysates from minimal FNA samples can be used for protein and RNA profiling of breast lesions without extensive sample processing. The high sensitivity of the two technologies, PEA and NS, allowed us to analyze a large number of proteins and mRNA in almost all QC‐approved samples. Our results demonstrate that the molecular profiling is reproducible and correlates well with results from routine IHC‐based analysis of ER, PGR, Ki67, and HER2. The findings indicate that FNA‐based analysis by NS technology may correlate better to IHC assessments compared to qPCR analysis of formalin‐fixed paraffin‐embedded CNB material (Sinn *et al*., 2017). Although our results are encouraging, independent, larger patient cohorts need to be analyzed to define cutoffs for future subtyping, and to evaluate clinical utility.

A significant correlation was observed between PEA‐based and NS‐based ERBB2 expression levels in BC samples (Fig. 3). Protein and/or mRNA analysis of ERBB2 in FNA samples may be sufficient to select samples for confirmation by FISH. Moreover, in three cases we observed major quantitative alterations of several proteins in *ex vivo* FNA samples taken postsurgery, compared to fresh FNA samples from the same patients at diagnosis 35–38 days earlier (Fig. S3). In one case, ERBB2 protein levels increased two‐ to fivefold after surgery compared with baseline. Furthermore, we observed a greater than 10‐fold increase in several immune‐related proteins (e.g., CD8A, CD5, IL‐8, CCL4, CXCL9, and MMP12). Whether this increase is a consequence of progression or an effect of surgery remains to be investigated. This result is interesting and in line with previous studies, providing arguments for protein analysis of fresh cytological material rather than postsurgery material (Bridge, 2017).

To investigate whether it would be possible to accurately discriminate between cancer and benign lesions using protein profiling of FNA samples, we modeled compact yet informative multiple regression signatures. A signature comprised of a surprisingly small number of proteins appeared to completely discriminate between cancer and benign lesions. The signature was independently validated using eight samples from seven patients where the initial cytological examination did not provide a conclusive diagnosis and whose biopsies were not used in identifying the signature. All these patients were correctly classified by the signature. Despite the low number of tested samples, these results are very promising and unlikely to be solely due to chance, which indicates the feasibility of rapid FNA diagnostics of malignancy via protein analysis. The ‘benign *vs*. cancer’ protein signature was based on the levels of a set of 11 proteins, and the first six proteins, briefly discussed below, contributed 80% of the predictive capacity of the model.

The first protein, the angiogenic factor FGFBP1, is a carrier protein for FGFs. It is expressed mainly in squamous epithelial cells, epidermal cells, and some types of glandular cells, but shows weak or negative staining in BC (http://www.proteinatlas.org). We observed significantly higher levels of FGFBP1 in benign lesions compared to cancer. The relatively high level of this protein in fibroadenomas has, to our knowledge, not been described previously. We also observed increased levels of FGF2 and EGF in fibroadenomas (data not shown). These observations contrast with other reports where FGFBP1 mRNA levels increased in postsurgery samples of cancer compared to normal tissue (Kagan *et al*., 2003). However, our results indicate increased protein levels in ER‐negative compared to ER‐positive subtypes (Table S4 with figures).

The second contributor to the signature was the matrix protein decorin (DCN), a proteoglycan highly expressed in stroma but not in cancer (Järvinen and Prince, 2015). Our observations confirm a negative correlation with more aggressive subtypes (Fig. S5).

The third protein of the signature was the pro‐protein convertase furin, a protease known to activate a number of cancer related substrates (Bassi *et al*., 2005). In agreement with our observations, recent studies strengthen the role of furin in cancer progression (Jaaks and Bernasconi, 2017).

The fourth protein was the pro‐angiogenic, anti‐inflammatory, antimetastatic enzyme heme oxygenase 1 (HO‐1 or hsp32), which is overexpressed in many cancers including BC (Duus Hjortso and Hald Andersen, 2014). Although HO‐1 was not significantly associated with malignancy by univariate analysis ‘cancer *vs*. benign’, it contributed to the signature and its levels correlated with those of the fifth member of the signature, GPNMB (Spearman rank *R* = 0.82).

High levels of GPNMB have been associated with TNB and increased risk of recurrence, and the protein represents an emerging target for immunotherapy (Maric *et al*., 2013; Zhou *et al*., 2012). We did not observe significant increases in more aggressive subtypes of BC, however.

The immune‐related chemokine CXCL9 was the sixth signature protein, and it is increased in many cancers (Ding *et al*., 2016). For instance, its transcript levels were increased in FNA samples from BC compared to benign lesions (André *et al*., 2009). Moreover, expression of CXCL9 has been associated with tumor‐infiltrating lymphocytes (TILs) and response to neoadjuvant chemotherapy in BC (Denkert *et al*., 2010). Interestingly, we observed not only an overall increase in CXCL9 in cancer, but also a significant increase in ER‐negative subtypes compared to luminal A cancers, and these levels correlated significantly with levels of the TIL‐related markers CD8A, CD4, and CD5. In addition, we observed differential expression of several other immune‐related proteins between BC subtypes (to be published elsewhere).

## 5 Conclusions

Our results show that PEA‐based protein profiles in FNA samples may allow reliable distinction of cancer and benign lesions and thus provide support for a conclusive diagnosis, which would be very important for early diagnosis of BC. In addition, PEA and/or NS‐based analysis of FNA samples may prove valuable for subtyping of BC, for therapy selection, and for monitoring the course of disease and responses to therapy by minimally invasive samples. In our view, the FNA material accurately represents the typical heterogeneous population of cells from the tumor at a given moment. PEA profiling of cytology material provides a ‘snapshot’ of the microenvironment of the tumor as the levels of most of the proteins and mRNA correlate well with the corresponding proteins in routine IHC from parallel CNB samples. Moreover, the preliminary data on alterations postsurgery are interesting and deserve further research of changes related to the microenvironment shortly after surgical removal of the tumor. We next aim to validate these findings in independent cohorts with prospective observation.

## Conflict of interest

UL is founder and shareholder of Olink Proteomics. LW is currently employed by Olink Proteomics but was not employed by this company at the time when she contributed to this study. No other authors declare any conflict of interests.

## Author contributions

BF, MKM, TH, RL, UL, and GA were responsible for project design and infrastructure. JK was responsible for patient selection and sampling of material. GA, SB, BF, MKM, LW, AE, and NRM were responsible for pretesting of sample preparation and experimental conditions. BF and SB were responsible for sample collection and sample preparation. AA was responsible for data analysis, statistics, and bioinformatics. BF was responsible for writing the manuscript with contributions by all co‐authors.

## Supporting information


**Fig. S1.** Flowchart for PEA analysis. Overview of all FNA *leftover* samples, exclusion of samples due to quality criteria, sample preparation, PEA analysis, and final data analysis.
**Fig. S2.** A weak correlation was observed between success rate of PEA results and pellet size.
**Fig. S3.** (a‐f). Protein profiles of postsurgery vs. presurgery FNA samples.
**Fig. S4.** (a‐f). Protein profiles: intra‐ and interpatient variation.
**Fig. S5.** Significantly different expression of four proteins from the 11‐protein signature.
**Table S1.** All samples for PEA and diagnosis by cytology (FNA material). Overview of all 58 patients, samples and final patient diagnoses (benign (*n* = 33) and cancer (*n* = 25) subtypes) according to IHC.
**Table S2.** Benign samples. In total, 33 patients were included and analyzed by PEA.
**Table S3.** All cancer samples from a total of 25 patients.
**Table S4.** Protein levels of FGFBP1.
**Table S5.** Proteins in the PEA panels used.
