# Supplementary material for: A fine‐needle aspiration‐based protein signature discriminates benign from malignant breast lesions
Source: Mol Oncol. 2018 Aug 9;12(9):1415–28. doi: 10.1002/1878-0261.12350 (PMC6120227; doi:10.1002/1878-0261.12350)
Supplement: Supplementary file 1 — Fig. S1. Flowchart for PEA analysis. Overview of all FNA leftover samples, exclusion of samples due to quality criteria, sample preparation, PEA analysis, and final data analysis. Fig. S2. A weak correlation was observed between success rate of PEA results and pellet size. Fig. S3. (a‐f). Protein profiles of postsurgery vs. presurgery FNA samples. Fig. S4. (a‐f). Protein profiles: intra‐ and interpatient variation. Fig. S5. Significantly different expression of four proteins from the 11‐protein signature. Table S1. All samples for PEA and diagnosis by cytology (FNA material). Overview of all 58 patients, samples and final patient diagnoses (benign (n = 33) and cancer (n = 25) subtypes) according to IHC. Table S2. Benign samples. In total, 33 patients were included and analyzed by PEA. Table S3. All cancer samples from a total of 25 patients. Table S4. Protein levels of FGFBP1. Table S5. Proteins in the PEA panels used. [file MOL2-12-1415-s001.docx]

**Supplementary Figures and Tables**

**Supplementary Figure 1.** **Flow chart for PEA analysis.** Overview of all FNA *leftover* samples, exclusion of samples due to quality criteria, sample preparation, PEA analysis and final data analysis. Twenty-two of the 34 cancer samples were selected for NanoString analysis.

92 Samples

84

77

74 Samples for PEA

73

72

8 Samples were excluded as they failed to meet the *cytology* quality control (QC) criteria by not being representative, having very low cellularity, or a high blood contamination (QC by microscopy).

7 Samples were excluded due to lack of visible material after cell preparation (cell pellets below 0.5 µL).

3 Samples were excluded because of insufficient
 protein concentrations (<0.5 mg/mL).

1 Sample (FD46 from patient #139) was excluded as it failed to meet PEA assay internal QC criteria.

1 Sample (FD82) excluded. The sample was originally thought to represent a lymph node metastasis, but was shown to be normal tissue (Supplementary Table 2)

38
Samples

”Benign”

34
Samples
”Cancer”

72 PEA samples were selected for *data analysis*: 38 samples from 33 patients with benign lesions and 34 samples from 25 cancer patients.

**Supplementary Figure 2.** Correlation between success rate of PEA results and pellet size.

A weak correlation was observed between success rate of PEA results and pellet size. >40% of all proteins in the PEA panels were detectable and above limit of detection (LOD; *i.e.* no missing values, or NAs, defined as “not a number”) in FNA-samples, e.g. ERBB2 was detected in 78 of 79 samples. We estimated the size of cell pellets according to an arbitrary 3-level scale. After protein determination, pellet "size 1" correspond to a median 36 µg protein (range 10-100 µg), pellet "size 2" correspond to a median 148 µg protein (range 100-300 µg) pellet "size 3" correspond to a median 332 µg protein (range 200-1,000 µg). In the figure to the right, estimated pellet size = 1 refers to a visible pellet but < 2 µL volume, the estimated pellet size = 2 corresponds to a volume of 2-5 µL, while an estimated pellet size = 3, corresponds to >5 µL volume. The upper row show data representing the immune-oncology PEA panel (ImmunoOncI) and the lower row show data representing the oncology II PEA panel (OncII).


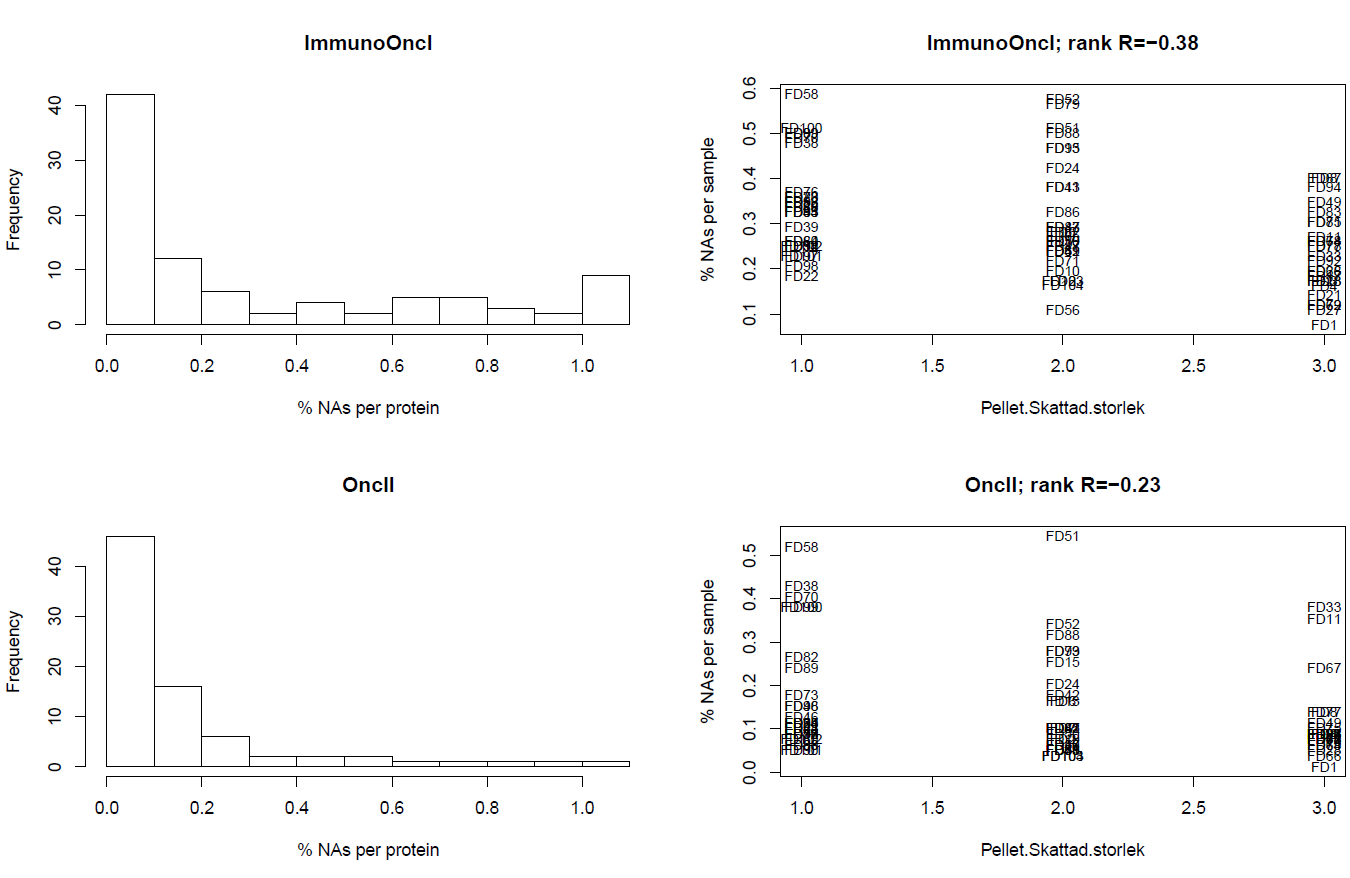


Estimated pellet size [arbitrary units]

Estimated pellet size [arbitrary units]

**Supplementary Figure 3 (a-f).** Scatter plots show protein levels (normalized protein concentration (NPX-values) in a 2-log scale) representing biological replicates compared to corresponding pre-surgery samples. Biological replicates of FNA-samples were taken post-surgery (a, b, and c) and scatter plots were also compared to pre-surgery samples (FD59, FD70 and FD24) from same patients (d, e and f). Results show decreased correlations (R) between pre- and post-surgery samples compared to replicate post-surgery material (R [replicates (0.96)] > R [pre- *vs.* post-surgery (0.85)]).

**Supplementary Figure 4 (a-f).** Protein profiles of intra- & inter-patient variation (cytological material, FNA-samples). Apparently, in the perspective of Figure 2, intra-patient correlation is higher than that of “pre- *vs.* post-surgery” (within same patient) and, in turn, higher than the inter-patient variability. In summary: R [replicates (0.96)] > R [Intra-pat. (0.92-0.94)] > R [pre- vs post-surgery (0.85)] > R [Inter-pat. (0.71)]. Scatter plots show PEA data plotted pairwise along the X and Y axes. Expression levels of each protein were reported as normalized protein concentration (NPX-values) on a 2-log scale.

**Supplementary Figure 5.** Significantly different expression of proteins from the 11-protein signature. N equals the number of analysed samples in both panels. Two of the proteins, decorin (DCN) and the chemokine CXCL9 also differ significantly between subtypes of BC (Fig.5a). The analysis of correlation between the protein expression profiles and clinical, immunohistochemical, and technical variables provided raw p-values (Fig.5b). Figures are highlighted in red if significant after Bonferroni correction, otherwise left in black. Expression levels on the Y-axis of each protein were reported as normalized protein concentration (NPX-values) on a 2-log scale.

**Fig.5a**


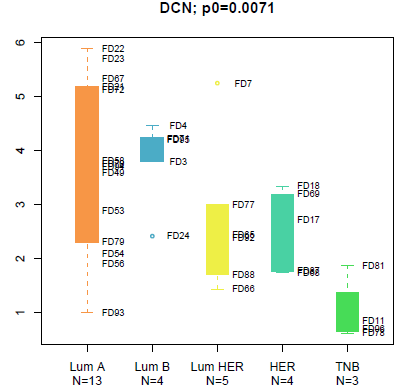

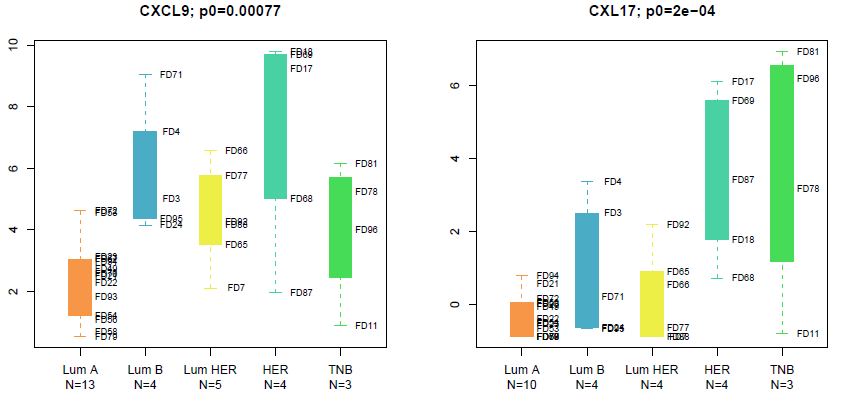


NPX Expression levels [2Log]

**Fig.5b**


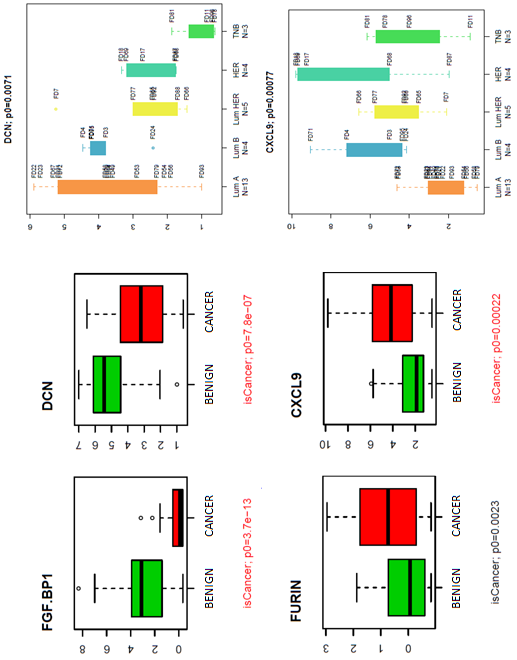


**Supplementary Table 1.** All samples subjected to PEA and diagnosis by cytology (FNA material). Overview of all 58 patients, samples and final patient diagnoses (benign (n=33) and cancer (n=25) subtypes) according to IHC. Different shades of grey indicate multiple samples from the same patient (for details, see Tables 2 and 3). In three cases (#113, #115 and #117), axillary metastases (Ax. Met.) were sampled and all three cytology samples showed high concentration of cancer cells. In two cases, benign and cancer samples were obtained from the same patient (pat. # 108 and #123). Text in *italics* *[within brackets]* indicate samples where final diagnosis was obtained for a given patient based on a parallel sample (multifocal tumours). In cases where multiple samples were obtained from a given patient, cytology and histology showed very high degrees of similarity. Therefore, we assign here the same cancer subtype to the parallel samples although the IHC analysis was performed on only one of the samples.

| **Patient  No.** | **Sample FD code** | **Final pat. diagnosis** |
| --- | --- | --- |
| 101 | 12 | Benign |
| 101 | 13 | Benign |
| 102 | 24 | Lum B |
| 103 | 58 | *[Lum A, Lobular]* |
| 103 | 79 | Lum A, Lobular |
| 104 | 01 | Benign |
| 104 | 02 | Benign |
| 105 | 03 | *[Lum B]* |
| 105 | 04 | Lum B |
| 106 | 06 | Benign |
| 107 | 07 | Lum HER2 |
| 108 | 10 | Benign (FA) |
| 108 | 11 | TNB |
| 109 | 17 | HER2 |
| 109 | 18 | *[HER2]* |
| 110 | 21 | Lum A |
| 110 | 22 | *[Lum A]* |
| 110 | 23 | *[Lum A]* |
| 112 | 49 | Lum A |
| 113 | 53 | *[Lum A] Ax. Met* |
| 113 | 54 | Lum A |
| 114 | 56 | Lum A (DCIS) |
| 115 | 65 | Lum HER2 |
| 115 | 66 | *[Lum HER2] Ax. Met* |
| 116 | 67 | Lum A |
| 117 | 68 | *[HER2] Ax. Met.* |
| 117 | 69 | HER2 |
| 118 | 70 | Lum A, lobular |
| 119 | 71 | Lum B |
| 120 | 72 | Lum A |
| 121 | 77 | Lum HER2 |
| 122 | 78 | TNB |
| 123 | 81 | TNB |
| 123 | 82 # | Normal lymph node (benign) |
| 124 | 87 | HER2 |
| 125 | 88 | Lum HER2 |
| 126 | 92 | Lum HER2 |
| **Patient  No.** | **Sample FD code** | **Final pat. Diagnosis** |
| 127 | 93 | Lum A |
| 127 | 94 | *[Lum A]* |
| 128 | 95 | Lum B |
| 129 | 96 | TNB |
| 130 | 08 | Benign |
| 131 | 15 | Benign |
| 132 | 19 | Benign |
| 132 | 20 | Benign |
| 133 | 27 | Benign |
| 134 | 28 | Benign |
| 135 | 33 | Benign |
| 136 | 38 | Benign |
| 137 | 39 | Benign |
| 138 | 41 | Benign |
| 140 | 43 | Benign |
| 140 | 44 | Benign |
| 141 | 47 | Benign |
| 141 | 48 | Benign |
| 142 | 50 | Benign |
| 143 | 51 | Benign |
| 144 | 52 | Benign |
| 145 | 59 | Benign |
| 146 | 60 | Benign |
| 147 | 61 | Benign |
| 148 | 62 | Benign |
| 149 | 63 | Benign |
| 150 | 64 | Benign |
| 151 | 73 | Benign |
| 152 | 74 | Benign |
| 153 | 75 | Benign |
| 154 | 76 | Benign |
| 155 | 80 | Benign |
| 156 | 83 | Benign |
| 157 | 86 | Benign |
| 158 | 89 | Benign |
| 158 | 90 | Benign |
| *# Sample FD82 was excluded from data analysis* | | |

**Supplementary Table 2.** Benign samples. In total, 33 patients were included and analyzed by PEA. From each of 6 patients, 2 samples were obtained (multifocal lesions, grey shaded rows) and in 2 patients (#108 and #123, FD code marked by *) we obtained both benign and cancer samples (compare Tab. 4 and 5). N.d. equals “not determined”.

| **Sample FD code** | **Patient  No.** | **Patient Age** | **Lesion side** | **Mam. code** | **First Diagnosis** | **Final diagnosis** | **Other diagnostic information** | | |
| --- | --- | --- | --- | --- | --- | --- | --- | --- | --- |
|  |  |  |  |  |  |  | **Fibro-adenoma** | **Fibro- adenosis** | **Hyperplasia** |
| 1 | 104 | 25 | Dex | n.d. | Benign | Benign | Yes |  |  |
| 2 | 104 | 25 | Sin | n.d. | Benign | Benign | Yes |  |  |
| 6 | 106 | 21 | Dex | 2 | Benign | Benign | Yes |  | Yes |
| 8 | 130 | 19 | Dex | 2 | Benign | Benign | Yes |  |  |
| 10* | 108 | 40 | Sin | 2 | Benign | Benign | Yes |  |  |
| 12 | 101 | 38 | Dex | 4 | Benign | Benign |  | Yes | Yes |
| 13 | 101 | 38 | Dex | 3 | Benign | Benign |  | Yes | Yes |
| 15 | 131 | 28 | Dex | 2 | Benign | Benign | Yes |  |  |
| 19 | 132 | 36 | Dex | 2 | Benign | Benign |  | Yes |  |
| 20 | 132 | 36 | Sin | 2 | Benign | Benign | Yes | Yes |  |
| 27 | 133 | 37 | Sin | 2 | **Suspect malign** | Benign | Yes (CNB) |  | Yes  (CNB) |
| 28 | 134 | 20 | Sin | 2 | Benign | Benign | Yes |  |  |
| 33 | 135 | 22 | Sin | 2 | Benign | Benign |  |  |  |
| 38 | 136 | 28 | Sin | 2 | Benign | Benign |  |  |  |
| 39 | 137 | 20 | Sin | 2 | Benign | Benign | Yes |  | Yes |
| 41 | 138 | 36 | Sin | 3 | Benign | Benign | Yes |  |  |
| 43 | 140 | 25 | Sin | 2 | Benign | Benign |  |  |  |
| 44 | 140 | 25 | Dex | 2 | Benign | Benign | Yes |  |  |
| 47 | 141 | 31 | Sin | 3 | Benign | Benign |  | Yes | Yes |
| 48 | 141 | 31 | Sin | 2 | Benign | Benign |  |  | Yes |
| 50 | 142 | 21 | Dex | n.d. | Benign | Benign | Yes |  |  |
| 51 | 143 | 32 | Sin | 2 | Benign | Benign | Yes (CNB) |  |  |
| 52 | 144 | 33 | Dex | 2 | Benign | Benign | Yes |  |  |
| 59 | 145 | 18 | Dex | 2 | Benign | Benign | Yes |  |  |
| 60 | 146 | 25 | Sin | 2 | Benign | Benign | Yes |  |  |
| 61 | 147 | 50 | Dex | 2 | Benign | Benign | Yes |  |  |
| 62 | 148 | 25 | Sin | 2 | Benign | Benign | Yes |  | Yes |
| 63 | 149 | 38 | Sin | 2 | Benign | Benign | Yes (CNB) |  |  |
| 64 | 150 | 32 | Dex | 2 | Benign | Benign | Yes |  |  |
| 73 | 151 | 31 | Sin | 2 | Benign | Benign | Yes |  |  |
| 74 | 152 | 33 | Dex | 2 | Benign | Benign | Yes |  |  |
| 75 | 153 | 52 | Sin | 5 | **Suspect malign** | Benign (Papilloma) |  |  |  |
| 76 | 154 | 18 | Dex | 2 | Benign | Benign | Yes |  |  |
| 80 | 155 | 51 | Sin | 3 | Benign | Benign | Yes |  |  |
| 82 | 123 | 57 | Axill | 5 | Suspect malign | Normal |  |  |  |
| 83 | 156 | 33 | Sin | 2 | Benign | Benign | Yes |  |  |
| 86 | 157 | 19 | Dex | 2 | Benign | Benign | Yes |  |  |
| 89 | 158 | 51 | Sin | 2 | Benign | Benign | Yes |  |  |
| 90 | 158 | 51 | Dex | 2 | Benign | Benign | Yes |  |  |

**Supplementary Table 3.** Cancer samples from a total of 25 patients. From 8 patients 2-3 samples were obtained per patient (marked by grey shaded rows): Four of these patients (#105, #109, #127 and #110) had multifocal lesions, 3 patients (#113, #115 and #117) had primary cancer and axillary metastases (rich in cancer cells according to cytology) and from one patient (#103) FNA sampling was repeated 11 days after the first sample. From 2 patients (#108 and #123), benign and cancer samples were obtained. For most cases, final diagnosis and IHC was based on CNB samples obtained directly after FNA sampling. In cases marked by “¤” final diagnosis and IHC was based on post-surgery sampling. (Compare Tables 2, 3 and 4). Median tumor size was 20 mm, multifocal lesions and metastases included, and 20 samples were from tumors <20 mm, nine samples from tumors 20-40 mm and five samples from tumors >40 mm (according to mammography and/or ultra-sound) in diameter. The symbol @ represents axillary metastasis (Met.).

| **Patient  No.** | **Sample FD code** | **Patient Age** | **Lesion  side** | **Mam.  code** | **Tumor  size [mm]** | **First  Diagnosis** | **Final  diagnosis** | **Histo.  grade** | **Her2 status** | **FISH  Amp.** | **ER  (%)** | **KI67 (%)** | **PGR  (%)** | **IHC Subtype** |
| --- | --- | --- | --- | --- | --- | --- | --- | --- | --- | --- | --- | --- | --- | --- |
| 105 | 03 | 36 | Dex | 5 | 45 | Cancer | IDC | 3 | 1+ | N.D. | 40 | 45 | 0 | Lum B |
| 105 | 04 | 36 | Dex | 5 | 22 | Cancer | IDC |  |  |  |  |  |  |  |
| 107 | 07 | 67 | Dex | 5 | 13 | **Susp. Malign** | IDC | N.D. | 2+ | No | 95 | 5 | 65 | Lum HER |
| 108 | 11 | 40 | Dex | 2 | 12 | **Susp. Malign** | IDC | 3 | 0 | N.D. | 0 | 60 | 5 | TNB |
| 109 | 17 | 57 | Sin | 5 | 19 | Cancer | IDC | 3 | 3+ | Yes | 0 | 78 | 0 | HER |
| 109 | 18 | 57 | Sin | 5 | 17 | Cancer | IDC |  |  |  |  |  |  |  |
| 110 | 21 | 53 | Sin | 4 | 19 | Cancer | IDC | 2 | 1+ | N.D. | 100 | 20 | 5 | Lum A |
| 110 | 22 | 53 | Sin | 4 | 14 | Cancer | IDC |  |  |  |  |  |  |  |
| 110 | 23 | 53 | Sin | 4 | 17 | Cancer | IDC |  |  |  |  |  |  |  |
| 102 | 24 | 67 | Sin | 5 | 20 | Cancer | IDC | 2 | 1+ | N.D. | 100 | 28 | 100 | Lum B |
| 112 | 49 | 67 | Sin | 5 | 27 | Cancer | IDC | 2 | 1+ | N.D. | 100 | 22 | 60 | Lum A |
| 113 | 53 | 83 | Dex @ | 5 | 10 | Cancer | IDC (Met.) |  |  |  |  |  |  |  |
| 113 | 54 | 83 | Dex | 5 | 18 | Cancer | IDC | 2 | 0 | N.D. | 100 | 13 | 80 | Lum A |
| 114 | 56 | 77 | Dex | 4 | 13 | **Susp. Malign** | DCIS (tubular) | 1 | 0 | N.D. | 100 | 11 | 100 | Lum A |
| 103 | 58 | 69 | Sin | 3 | 12 | **Unclear** | ILC |  |  |  |  |  |  |  |
| 115 | 65 | 41 | Sin | 5 | 40 | Cancer | IDC | 2 | 2+ | No | 100 | 30 | 85 | Lum HER |
| 115 | 66 | 41 | Sin @ | 5 | 14 | Cancer | IDC (Met.) |  |  |  |  |  |  |  |
| 116 | 67 | 76 | Dex | 5 | 32 | **Susp. Malign** | IDC | 2 | 0 | N.D. | 100 | 5 | 50 | Lum A |
| 117 | 68 | 46 | Sin @ | 5 | 30 | Cancer | IDC (Met.) |  |  |  |  |  |  |  |
| 117 | 69 | 46 | Sin | 5 | 85 | Cancer | IDC | 3 | 3+ | Yes | 10 | 40 | 0 | HER |
| 118 | 70 | 48 | Sin | 4 | 60 | Cancer | ILC | 2 | 0 | N.D. | 95 | 10 | 100 | Lum A |
| 119 | 71 | 48 | Sin | 5 | 45 | Cancer | IDC | 2 | 0 | N.D. | 95 | 30 | 80 | Lum B |
| 120 | 72 | 43 | Sin | 5 | 15 | Cancer | IDC | 1 | 0 | N.D. | 100 | 13 | 100 | Lum A |
| 121 | 77 | 86 | Dex | 5 | 20 | Cancer | IDC | 2 | 2+ | Yes | 100 | 29 | 90 | Lum HER |
| 122 | 78 | 52 | Sin | 5 | 25 | Cancer | IDC | 3 | 0 | N.D. | 2 | 60 | 0 | TNB |
| 103 | 79 | 69 | Sin | 4 | 8 | **Susp. Malign** | ILC | 2 | 0 | N.D. | 100 | 3 | 0 | Lum A |
| 123 | 81 | 58 | Dex | 5 | 40 | Cancer | IDC | 2 | 1+ | N.D. | 0 | 65 | 0 | TNB |
| 124 | 87 ¤ | 71 | Dex | 5 | 70 | Cancer | IDC/DCIS | 3 | 3+ | Yes | 0 | 35 | 0 | HER |
| 125 | 88 | 17 | Sin | 5 | 20 | Cancer | IDC | 3 | 2+ | No | 100 | 30 | 0 | Lum HER |
| 126 | 92 | 71 | Sin | 5 | 20 | Cancer | IDC | 3 | 2+ | No | 100 | 35 | 80 | Lum HER |
| 127 | 93 | 42 | Sin | 5 | 20 | Cancer | IDC/DCIS | 1 | 0 | N.D. | 100 | 15 | 100 | Lum A |
| 127 | 94 | 42 | Sin | 5 | 10 | Cancer | IDC |  |  |  |  |  |  |  |
| 128 | 95 | 89 | Dex | 5 | 38 | Cancer | IDC | 2 | 1+ | N.D. | 100 | 24 | 95 | Lum B |
| 129 | 96 | 64 | Sin | 5 | 40 | Cancer | IDC | 3 | 1+ | N.D. | 0 | 60 | 10 | TNB |

**Supplementary Table 4 and figures complementary to Table 4.** Protein levels of FGFBP1.

**Supplementary Table 5.** Proteins in the PEA-panels used (for more information: www.olink.com)

| **Protein name** | **Gene name** | **UniProt Accession** | **UniProt/ SwissProt ID** | **PEA panel Oncology II** | **PEA panel Immune_ Oncology I** | **Excluded proteins from  model (>25% NAN)** |
| --- | --- | --- | --- | --- | --- | --- |
| Tyrosine-protein kinase ABL1 | ABL1 | P00519 | ABL1_HUMAN | X |  |  |
| Adenosine deaminase | ADA | P00813 | ADA_HUMAN |  | X |  |
| Disintegrin and metalloproteinase domain-containing protein 8 (ADAM 8) | ADAM8 | P78325 | ADAM8_HUMAN | X |  |  |
| A disintegrin and metalloproteinase with thrombospondin motifs 15 (ADAM-TS 15) | ADAMTS15 | Q8TE58 | ATS15_HUMAN | X |  |  |
| Adhesion G-protein coupled receptor G1 | ADGRG1 | Q9Y653 | AGRG1_HUMAN |  | X | X |
| Angiopoietin-1 (ANG-1) | ANGPT1 | Q15389 | ANGP1_HUMAN |  | X |  |
| Angiopoietin-2 (ANG-2) | ANGPT2 | O15123 | ANGP2_HUMAN |  | X | X |
| Annexin A1 | ANXA1 | P04083 | ANXA1_HUMAN | X |  |  |
| Amphiregulin (AR) | AREG | P15514 | AREG_HUMAN | X |  |  |
| Arginase-1 | ARG1 | P05089 | ARGI1_HUMAN |  | X |  |
| Carbonic anhydrase 9 | CA9 | Q16790 | CAH9_HUMAN | X | X | X |
| Caspase-8 (CASP-8) | CASP8 | Q14790 | CASP8_HUMAN |  | X |  |
| C-C motif chemokine 13 | CCL13 | Q99616 | CCL13_HUMAN |  | X |  |
| C-C motif chemokine 17 | CCL17 | Q92583 | CCL17_HUMAN |  | X |  |
| C-C motif chemokine 19 | CCL19 | Q99731 | CCL19_HUMAN |  | X |  |
| C-C motif chemokine 2 | CCL2 | P13500 | CCL2_HUMAN |  | X |  |
| C-C motif chemokine 20 | CCL20 | P78556 | CCL20_HUMAN |  | X |  |
| C-C motif chemokine 23 | CCL23 | P55773 | CCL23_HUMAN |  | X |  |
| C-C motif chemokine 3 | CCL3 | P10147 | CCL3_HUMAN |  | X |  |
| C-C motif chemokine 4 | CCL4 | P13236 | CCL4_HUMAN |  | X |  |
| C-C motif chemokine 7 | CCL7 | P80098 | CCL7_HUMAN |  | X | X |
| C-C motif chemokine 8 | CCL8 | P80075 | CCL8_HUMAN |  | X |  |
| CD160 antigen | CD160 | O95971 | BY55_HUMAN | X |  |  |
| C-type lectin domain family 4 member K | CD207 | Q9UJ71 | CLC4K_HUMAN | X |  | X |
| Natural killer cell receptor 2B4 | CD244 | Q9BZW8 | CD244_HUMAN |  | X |  |
| CD27 antigen | CD27 | P26842 | CD27_HUMAN | X | X |  |
| Programmed cell death 1 ligand 1 (PD-L1) | CD274 | Q9NZQ7 | PD1L1_HUMAN |  | X | X |
| T-cell-specific surface glycoprotein CD28 | CD28 | P10747 | CD28_HUMAN |  | X |  |
| T-cell surface glycoprotein CD4 | CD4 | P01730 | CD4_HUMAN |  | X |  |
| Tumor necrosis factor receptor superfamily member 5 | CD40 | P25942 | TNR5_HUMAN |  | X |  |
| CD40 ligand (CD40-L) | CD40LG | P29965 | CD40L_HUMAN |  | X |  |
| CD48 antigen | CD48 | P09326 | CD48_HUMAN | X |  |  |
| T-cell surface glycoprotein CD5 | CD5 | P06127 | CD5_HUMAN |  | X |  |
| CD70 antigen | CD70 | P32970 | CD70_HUMAN | X | X | X |
| CD83 antigen (hCD83) | CD83 | Q01151 | CD83_HUMAN |  | X |  |
| T-cell surface glycoprotein CD8 alpha chain | CD8A | P01732 | CD8A_HUMAN |  | X |  |
| Cyclin-dependent kinase inhibitor 1 | CDKN1A | P38936 | CDN1A_HUMAN | X |  |  |
| Carcinoembryonic antigen-related cell adhesion molecule 1 | CEACAM1 | P13688 | CEAM1_HUMAN | X |  |  |
| Carcinoembryonic antigen-related cell adhesion molecule 5 | CEACAM5 | P06731 | CEAM5_HUMAN | X |  | X |
| Carboxypeptidase E (CPE) | CPE | P16870 | CBPE_HUMAN | X |  |  |
| **(Cont. Tab 5)**  **Protein name** | **Gene name** | **UniProt Accession** | **UniProt/ SwissProt ID** | **PEA panel Oncology II** | **PEA panel Immune_ Oncology I** | **Excluded proteins from  model (>25% NAN)** |
| Cornulin | CRNN | Q9UBG3 | CRNN_HUMAN | X |  |  |
| Cytotoxic and regulatory T-cell molecule | CRTAM | O95727 | CRTAM_HUMAN |  | X | X |
| Macrophage colony-stimulating factor 1 (CSF-1) | CSF1 | P09603 | CSF1_HUMAN |  | X |  |
| Cathepsin L2 | CTSV | O60911 | CATL2_HUMAN | X |  |  |
| Fractalkine | CX3CL1 | P78423 | X3CL1_HUMAN |  | X |  |
| Growth-regulated alpha protein | CXCL1 | P09341 | GROA_HUMAN |  | X |  |
| C-X-C motif chemokine 10 | CXCL10 | P02778 | CXL10_HUMAN |  | X |  |
| C-X-C motif chemokine 11 | CXCL11 | O14625 | CXL11_HUMAN |  | X |  |
| Stromal cell-derived factor 1 (SDF-1) | CXCL12 | P48061 | SDF1_HUMAN |  | X | X |
| C-X-C motif chemokine 13 | CXCL13 | O43927 | CXL13_HUMAN | X | X |  |
| VEGF coregulated chemokine 1 | CXCL17 | Q6UXB2 | VCC1_HUMAN | X |  |  |
| C-X-C motif chemokine 5 | CXCL5 | P42830 | CXCL5_HUMAN |  | X |  |
| Interleukin-8 (IL-8) | CXCL8 | P10145 | IL8_HUMAN |  | X |  |
| C-X-C motif chemokine 9 | CXCL9 | Q07325 | CXCL9_HUMAN |  | X |  |
| Protein CYR61 | CYR61 | O00622 | CYR61_HUMAN | X |  |  |
| Decorin | DCN | P07585 | PGS2_HUMAN |  | X |  |
| Delta-like protein 1 | DLL1 | O00548 | DLL1_HUMAN | X |  |  |
| Interleukin-27 subunit beta (IL-27 subunit beta) | EBI3 | Q14213 | IL27B_HUMAN |  | X |  |
| Pro-epidermal growth factor (EGF) | EGF | P01133 | EGF_HUMAN | X | X |  |
| Ephrin type-A receptor 2 | EPHA2 | P29317 | EPHA2_HUMAN | X |  |  |
| Receptor tyrosine-protein kinase erbB-2 | ERBB2 | P04626 | ERBB2_HUMAN | X |  |  |
| Receptor tyrosine-protein kinase erbB-3 | ERBB3 | P21860 | ERBB3_HUMAN | X |  |  |
| Receptor tyrosine-protein kinase erbB-4 | ERBB4 | Q15303 | ERBB4_HUMAN | X |  |  |
| Endothelial cell-specific molecule 1 (ESM-1) | ESM1 | Q9NQ30 | ESM1_HUMAN | X |  |  |
| FAS-associated death domain protein | FADD | Q13158 | FADD_HUMAN | X |  |  |
| Tumor necrosis factor ligand superfamily member 6 | FASLG/FasL | P48023 | TNFL6_HUMAN | X | X |  |
| Fc receptor-like B | FCRLB | Q6BAA4 | FCRLB_HUMAN | X |  | X |
| Fibroblast growth factor 2 (FGF-2) | FGF2 | P09038 | FGF2_HUMAN |  | X |  |
| Fibroblast growth factor-binding protein 1 (FGF-BP) | FGFBP1 | Q14512 | FGFP1_HUMAN | X |  |  |
| Vascular endothelial growth factor receptor 3 (VEGFR-3) | FLT4 | P35916 | VGFR3_HUMAN | X |  | X |
| Folate receptor alpha (FR-alpha) | FOLR1 | P15328 | FOLR1_HUMAN | X |  |  |
| Folate receptor gamma (FR-gamma) | FOLR3 | P41439 | FOLR3_HUMAN | X |  |  |
| Furin | FURIN | P09958 | FURIN_HUMAN | X |  |  |
| Glypican-1 | GPC1 | P35052 | GPC1_HUMAN | X |  | X |
| Transmembrane glycoprotein NMB | GPNMB | Q14956 | GPNMB_HUMAN | X |  |  |
| Granzyme A | GZMA | P12544 | GRAA_HUMAN |  | X |  |
| Granzyme B | GZMB | P10144 | GRAB_HUMAN | X | X |  |
| Granzyme H | GZMH | P20718 | GRAH_HUMAN | X | X |  |
| Hepatocyte growth factor | HGF | P14210 | HGF_HUMAN | X | X |  |
| Heme oxygenase 1 (HO-1) | HMOX1 | P09601 | HMOX1_HUMAN |  | X |  |
| ICOS ligand | ICOSLG | O75144 | ICOSL_HUMAN | X | X | X |
| **(Cont. Tab 5)**  **Protein name** | **Gene name** | **UniProt Accession** | **UniProt/ SwissProt ID** | **PEA panel Oncology II** | **PEA panel Immune_ Oncology I** | **Excluded proteins from  model (>25% NAN)** |
| Interferon beta (IFN-beta) | IFNB1 | P01574 | IFNB_HUMAN |  | X | X |
| Interferon gamma (IFN-gamma) | IFNG | P01579 | IFNG_HUMAN |  | X | X |
| Interferon gamma receptor 1 (IFN-gamma receptor 1) | IFNGR1 | P15260 | INGR1_HUMAN | X |  |  |
| Insulin-like growth factor 1 receptor | IGF1R | P08069 | IGF1R_HUMAN | X |  |  |
| Interleukin-10 (IL-10) | IL10 | P22301 | IL10_HUMAN |  | X | X |
| Interleukin-12 subunit alpha (IL-12A) | IL12A | P29459 | IL12A_HUMAN |  | X | X |
| Interleukin-12 subunit alpha (IL-12A) | IL12A | P29459 | IL12A_HUMAN |  | X | X |
| Interleukin-12 subunit beta (IL-12B) | IL12B | P29460 | IL12B_HUMAN |  | X |  |
| Interleukin-12 receptor subunit beta-1 (IL-12 receptor subunit beta-1) | IL12RB1 | P42701 | I12R1_HUMAN |  | X | X |
| Interleukin-13 (IL-13) | IL13 | P35225 | IL13_HUMAN |  | X | X |
| Interleukin-18 (IL-18) | IL18 | Q14116 | IL18_HUMAN |  | X |  |
| Interleukin-1 alpha (IL-1 alpha) | IL1A | P01583 | IL1A_HUMAN |  | X |  |
| Interleukin-2 (IL-2) | IL2 | P60568 | IL2_HUMAN |  | X | X |
| Interleukin-21 (IL-21) | IL21 | Q9HBE4 | IL21_HUMAN |  | X | X |
| Interleukin-33 (IL-33) | IL33 | O95760 | IL33_HUMAN |  | X |  |
| Interleukin-4 (IL-4) | IL4 | P05112 | IL4_HUMAN |  | X | X |
| Interleukin-5 (IL-5) | IL5 | P05113 | IL5_HUMAN |  | X | X |
| Interleukin-6 (IL-6) | IL6 | P05231 | IL6_HUMAN | X | X |  |
| Interleukin-7 (IL-7) | IL7 | P13232 | IL7_HUMAN |  | X | X |
| Integrin alpha-V | ITGAV | P06756 | ITAV_HUMAN | X |  |  |
| Integrin beta-5 | ITGB5 | P18084 | ITB5_HUMAN | X |  |  |
| Vascular endothelial growth factor receptor 2 (VEGFR-2) | KDR | P35968 | VGFR2_HUMAN | X | X |  |
| Kit ligand | KITLG | P21583 | SCF_HUMAN | X |  |  |
| Kallikrein-11 (hK11) | KLK11 | Q9UBX7 | KLK11_HUMAN | X |  |  |
| Kallikrein-13 | KLK13 | Q9UKR3 | KLK13_HUMAN | X |  |  |
| Kallikrein-14 (hK14) | KLK14 | Q9P0G3 | KLK14_HUMAN | X |  |  |
| Kallikrein-8 (hK8) | KLK8 | O60259 | KLK8_HUMAN | X |  |  |
| Natural killer cells antigen CD94 | KLRD1 | Q13241 | KLRD1_HUMAN |  | X | X |
| Lysosome-associated membrane glycoprotein 3 (LAMP-3) | LAMP3 | Q9UQV4 | LAMP3_HUMAN |  | X | X |
| Galectin-1 (Gal-1) | LGALS1 | P09382 | LEG1_HUMAN | X | X |  |
| Galectin-9 (Gal-9) | LGALS9 | O00182 | LEG9_HUMAN |  | X |  |
| T-lymphocyte surface antigen Ly-9 | LY9 | Q9HBG7 | LY9_HUMAN | X |  |  |
| Tyrosine-protein kinase Lyn | LYN | P07948 | LYN_HUMAN | X |  |  |
| Ly6/PLAUR domain-containing protein 3 | LYPD3 | O95274 | LYPD3_HUMAN | X |  |  |
| Midkine (MK) | MDK | P21741 | MK_HUMAN | X |  |  |
| Methionine aminopeptidase 2 (MAP 2) | METAP2 | P50579 | MAP2_HUMAN | X |  |  |
| Melanoma-derived growth regulatory protein | MIA | Q16674 | MIA_HUMAN | X |  |  |
| MHC class I polypeptide-related sequence A (MIC-A) | MICA | Q29983 | MICA_HUMAN | X | X | X |
| MHC class I polypeptide-related sequence B (MIC-B) | MICB | Q29980 | MICB_HUMAN | X | X |  |
| Macrophage metalloelastase (MME) | MMP12 | P39900 | MMP12_HUMAN |  | X |  |
| **(Cont. Tab 5)**  **Protein name** | **Gene name** | **UniProt Accession** | **UniProt/ SwissProt ID** | **PEA panel Oncology II** | **PEA panel Immune_ Oncology I** | **Excluded proteins from  model (>25% NAN)** |
| Matrilysin | MMP7 | P09237 | MMP7_HUMAN |  | X |  |
| Mesothelin | MSLN | Q13421 | MSLN_HUMAN | X |  | X |
| Mucin-16 (MUC-16) | MUC16 | Q8WXI7 | MUC16_HUMAN | X |  |  |
| Natural cytotoxicity triggering receptor 1 | NCR1 | O76036 | NCTR1_HUMAN |  | X | X |
| Nectin-4 | NECTIN4 | Q96NY8 | NECT4_HUMAN | X |  |  |
| Nitric oxide synthase, endothelial | NOS3 | P29474 | NOS3_HUMAN |  | X |  |
| 5'-nucleotidase (5'-NT) | NT5E | P21589 | 5NTD_HUMAN | X |  |  |
| Programmed cell death protein 1 (Protein PD-1) | PDCD1 | Q15116 | PDCD1_HUMAN |  | X | X |
| Programmed cell death 1 ligand 2 (PD-1 ligand 2) | PDCD1LG2 | Q9BQ51 | PD1L2_HUMAN |  | X | X |
| Platelet-derived growth factor subunit B (PDGF subunit B) | PDGFB | P01127 | PDGFB_HUMAN |  | X |  |
| Placenta growth factor (PlGF) | PGF | P49763 | PLGF_HUMAN |  | X |  |
| Podocalyxin | PODXL | O00592 | PODXL_HUMAN | X |  |  |
| Pancreatic prohormone | PPY | P01298 | PAHO_HUMAN | X |  | X |
| Pleiotrophin (PTN) | PTN | P21246 | PTN_HUMAN |  | X | X |
| Proto-oncogene tyrosine-protein kinase receptor Ret | RET | P07949 | RET_HUMAN | X |  |  |
| R-spondin-3 | RSPO3 | Q9BXY4 | RSPO3_HUMAN | X |  | X |
| Protein S100-A11 | S100A11 | P31949 | S10AB_HUMAN | X |  |  |
| Protein S100-A4 | S100A4 | P26447 | S10A4_HUMAN | X |  |  |
| Secretory carrier-associated membrane protein 3 (Secretory carrier membrane protein 3) | SCAMP3 | O14828 | SCAM3_HUMAN | X |  |  |
| Syndecan-1 (SYND1) | SDC1 | P18827 | SDC1_HUMAN | X |  |  |
| Seizure 6-like protein | SEZ6L | Q9BYH1 | SE6L1_HUMAN | X |  |  |
| Mothers against decapentaplegic homolog 5 (MAD homolog 5) | SMAD5 | Q99717 | SMAD5_HUMAN | X |  |  |
| SPARC | SPARC | P09486 | SPRC_HUMAN | X |  |  |
| T-cell leukemia/lymphoma protein 1A | TCL1A | P56279 | TCL1A_HUMAN | X |  |  |
| Angiopoietin-1 receptor | TEK | Q02763 | TIE2_HUMAN |  | X | X |
| Tissue factor pathway inhibitor 2 (TFPI-2) | TFPI2 | P48307 | TFPI2_HUMAN | X |  |  |
| Protransforming growth factor alpha | TGFA | P01135 | TGFA_HUMAN | X |  |  |
| Transforming growth factor beta-1 (TGF-beta-1) | TGFB1 | P01137 | TGFB1_HUMAN |  | X | X |
| TGF-beta receptor type-2 (TGFR-2) | TGFBR2 | P37173 | TGFR2_HUMAN | X |  |  |
| Toll-like receptor 3 | TLR3 | O15455 | TLR3_HUMAN | X |  |  |
| Tumor necrosis factor | TNF | P01375 | TNFA_HUMAN |  | X | X |
| Tumor necrosis factor receptor superfamily member 12A | TNFRSF12A | Q9NP84 | TNR12_HUMAN |  | X | X |
| Tumor necrosis factor receptor superfamily member 19 | TNFRSF19 | Q9NS68 | TNR19_HUMAN | X |  |  |
| Tumor necrosis factor receptor superfamily member 21 | TNFRSF21 | O75509 | TNR21_HUMAN |  | X |  |
| Tumor necrosis factor receptor superfamily member 4 | TNFRSF4 | P43489 | TNR4_HUMAN | X | X |  |
| Tumor necrosis factor receptor superfamily member 6B | TNFRSF6B | O95407 | TNF6B_HUMAN | X |  |  |
|  |  |  |  |  |  |  |
| **(Cont. Tab 5)**  **Protein name** | **Gene name** | **UniProt Accession** | **UniProt/ SwissProt ID** | **PEA panel Oncology II** | **PEA panel Immune_ Oncology I** | **Excluded proteins from  model (>25% NAN)** |
| Tumor necrosis factor receptor superfamily member 9 | TNFRSF9 | Q07011 | TNR9_HUMAN |  | X |  |
| Tumor necrosis factor ligand superfamily member 10 | TNFSF10 | P50591 | TNF10_HUMAN | X | X |  |
| Tumor necrosis factor ligand superfamily member 12 | TNFSF12 | O43508 | TNF12_HUMAN |  | X |  |
| Tumor necrosis factor ligand superfamily member 13 | TNFSF13 | O75888 | TNF13_HUMAN | X |  |  |
| Tumor necrosis factor ligand superfamily member 14 | TNFSF14 | O43557 | TNF14_HUMAN |  | X |  |
| TNF-related apoptosis-inducing ligand | TRAIL | P50591 | TNF10_HUMAN | X | X |  |
| Alpha-taxilin | TXLNA | P40222 | TXLNA_HUMAN | X |  |  |
| Vascular endothelial growth factor A (VEGF-A) | VEGFA | P15692 | VEGFA_HUMAN | X | X |  |
| Vascular endothelial growth factor C (VEGF-C) | VEGFC | P49767 | VEGFC_HUMAN |  | X | X |
| WAP four-disulfide core domain protein 2 | WFDC2 | Q14508 | WFDC2_HUMAN | X |  |  |
| Wnt inhibitory factor 1 (WIF-1) | WIF1 | Q9Y5W5 | WIF1_HUMAN | X |  | X |
| Vimentin | VIM | P08670 | VIME_HUMAN | X |  |  |
| WNT1-inducible-signaling pathway protein 1 (WISP-1) | WISP1 | O95388 | WISP1_HUMAN | X |  |  |
| Xaa-Pro aminopeptidase 2 | XPNPEP2 | O43895 | XPP2_HUMAN | X |  | X |
